# Quantifying Facial Gestures Using Deep Learning in a New World Monkey

**DOI:** 10.1002/ajp.70013

**Published:** 2025-02-28

**Authors:** Filippo Carugati, Dayanna Curagi Gorio, Chiara De Gregorio, Daria Valente, Valeria Ferrario, Brice Lefaux, Olivier Friard, Marco Gamba

**Affiliations:** ^1^ Department of Life Sciences and Systems Biology Università di Torino Torino Italy; ^2^ Department of Psychology University of Warwick Coventry UK; ^3^ Parco Natura Viva Garda Zoological Park Bussolengo Italy; ^4^ Chester Zoo Chester UK; ^5^ Zoo de Mulhouse Mulhouse France

**Keywords:** cotton‐top tamarin, DeepLabCut, markerless pose estimation, primate face, *Saguinus oedipus*

## Abstract

Facial gestures are a crucial component of primate multimodal communication. However, current methodologies for extracting facial data from video recordings are labor‐intensive and prone to human subjectivity. Although automatic tools for this task are still in their infancy, deep learning techniques are revolutionizing animal behavior research. This study explores the distinctiveness of facial gestures in cotton‐top tamarins, quantified using markerless pose estimation algorithms. From footage of captive individuals, we extracted and manually labeled frames to develop a model that can recognize a custom set of landmarks positioned on the face of the target species. The trained model predicted landmark positions and subsequently transformed them into distance matrices representing landmarks' spatial distributions within each frame. We employed three competitive machine learning classifiers to assess the ability to automatically discriminate facial configurations that cooccur with vocal emissions and are associated with different behavioral contexts. Initial analysis showed correct classification rates exceeding 80%, suggesting that voiced facial configurations are highly distinctive from unvoiced ones. Our findings also demonstrated varying context specificity of facial gestures, with the highest classification accuracy observed during yawning, social activity, and resting. This study highlights the potential of markerless pose estimation for advancing the study of primate multimodal communication, even in challenging species such as cotton‐top tamarins. The ability to automatically distinguish facial gestures in different behavioral contexts represents a critical step in developing automated tools for extracting behavioral cues from raw video data.

## Summary

- Markerless pose estimation can represent a promising tool for quantifying primate facial gestures.
- Facial configurations associated with vocal emissions are highly distinctive.
- Cotton‐top tamarins present a different context‐specificity of facial gestures across different behavioral classes.

## Graphical abstract

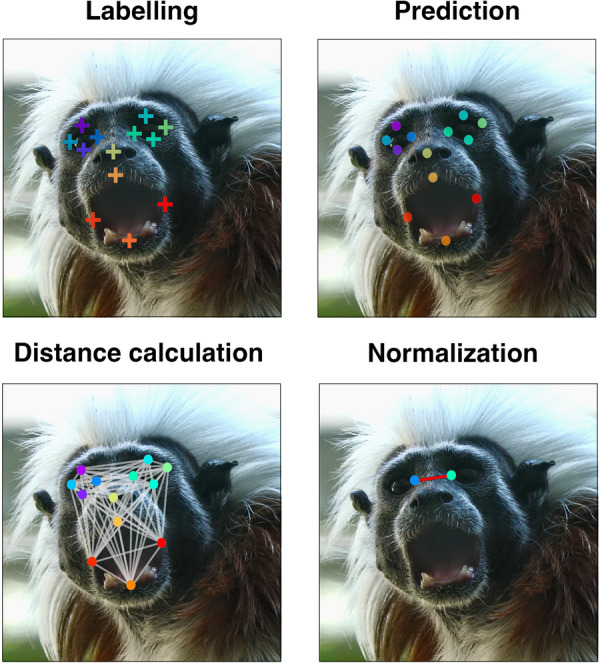

## 1 Introduction

Visual communication plays a crucial role in social interactions among primates (Waller et al. 2022). Both humans and other primates exhibit a wide range of facial expressions that convey emotional states and intentions within their social groups (Kret et al. 2020). Facial gestures are essential in visual communication, facilitating interactions in various contexts, from aggression to affiliation (Mitchell et al. 2014), and allowing fine modulation of behavioral interactions between group members or individuals from different groups (Maestripieri 1997; Parr et al. 2005; Gallo et al. 2022). Previous studies showed that primate brains have excellent connectivity between the motor and visual cortices (Molnár et al. 2014) and suggested that primates, more than other mammals, strongly rely on visual communication (Dobson and Sherwood 2011).

Facial gestures can carry subtle variations that indicate the specific communicative context a facial configuration conveys (Parr et al. 2005). These nuances can indicate aggression, dominance, submission, and appeasement (Petersen et al. 2018). For instance, while an open‐mouth display with exposed teeth commonly indicates submission (Maestripieri and Wallen 1997), the degree of tooth exposure during a yawn can suggest varying levels of aggression or social tension (Vick and Paukner 2010; Leone et al. 2014). The ability to modulate and interpret these signals is essential for maintaining social bonds, a hallmark of complex primate societies (Brügger et al. 2021).

Investigating the specificity of facial configurations across different behavioral contexts represents a crucial step toward understanding the role of visual signals in primates' communication. Although few studies—based on a few model species—have been conducted on this topic, most findings suggest that facial configuration often aligns closely with a specific behavioral context. For instance, Parr et al. (2005) demonstrated that chimpanzee facial expressions exhibit high context‐specificity, corresponding to seven behavioral macro‐categories identified through principal component analysis. Similarly, crested black macaques (*Macaca nigra*) adjust the morphological structure of silent‐bared teeth displays based on the context in which they are performed (Clark et al. 2020). Such context‐specificity in facial gestures has also been observed in other mammals, including cats (Scott and Florkiewicz 2023) and mice (Defensor et al. 2012).

The desire to go deeper into the complexity of animal communication systems compels us to consider which technologies currently available can most effectively facilitate advancements in this field. Several studies indicated effective quantitative methodologies for studying visual communication in mammals, but they rely on a visual, operator‐based screening. Facial Action Coding System (FACS) is a tool for analyzing and quantifying facial expressions in animals, particularly primates, allowing for a more precise and objective study of communication through facial expressions (Ekman and Friesen 1978; Waller et al. 2020). Although FACS has significantly advanced our study of facial expressions, this methodological approach requires a deep knowledge of the facial muscle structures of the species under study, effectively limiting the number of available models (Kaminski et al. 2019; Waller et al. 2020).

Moreover, the complexity of facial musculature also makes it complicated to identify and classify facial gestures in discrete and uniform categories, considering that facial configuration may subtly change and be shaped by interaction with conspecifics (Liebal et al. 2019).

Technological advancements in audiovisual quality and storage capacity over recent decades have created extensive video data sets (Janisch et al. 2021). Despite these resources, time constraints have limited the full utilization of the informational content, prompting a growing interest among researchers in leveraging artificial intelligence for automated video analysis (Luxem et al. 2023). Indeed, machine learning algorithms have come to play a pivotal role in analyzing audiovisual materials across various fields of research, including the study of animal behavior. These algorithms have been proven effective in discriminating between different individuals of the same species (Schofield et al. 2019; Guo et al. 2020; Paulet et al. 2024) based on facial features.

Among the different approaches for automatizing the extraction of behavioral cues from video data, markerless pose estimation has demonstrated its potential in capturing animal movements and linking them to specific behavior based on body posture, whether of the entire body or individual body parts. Researchers have successfully employed markerless pose estimation to track animals both in controlled or natural settings and to automatically extract information to describe their behaviors, as shown for crickets (Hayakawa et al. 2024), crayfishes (Suryanto et al. 2023), dolphins (Tseng et al. 2024), rats (Popik et al. 2024; Lapp et al. 2023), and primates (Fuchs et al. 2024).

Recently, researchers successfully used markerless pose estimation software to detect facial gestures in primates recorded in captivity and the wild. This study showed how it could automatically discriminate between facial configurations associated with vocalization emission from those silent (Carugati et al. 2025). Following this new approach, we selected a challenging model species in our study, the cotton‐top tamarin (*Saguinus oedipus*), to investigate whether Deep Learning technologies could detect particular facial key points and extract behavioral cues from the predicted key points. To identify a particular positioning of our key points, we alternatively used the terms “facial gesture” or “facial configuration,” without inferring about the intentionality or the communicative meaning of those displays. The cotton‐top tamarin is a petite primate from the New World weighing under 0.5 kg, and it is easily distinguished by its lengthy, white, sagittal crest from forehead to shoulders. Socially, cotton‐top tamarins exhibit a broad range of behaviors. Groups usually do not exceed 10 individual and have high territorial exclusivity (Savage et al. 1996; Washabaugh et al. 2002). These tamarins are known for their high cooperation and shared infant care (Cleveland and Snowdon 1984). Their communication methods include chemical, vocal, and visual signals, which may change across contexts and play a critical role in maintaining social stability (Snowdon et al. 1982). Both the morphological and behavioral features make cotton‐top tamarin a suitable model species for investigating the ability of deep learning methods to identify facial key points and extract information concerning the associated behavioral context. The tiny cranial size and the uniform blackish coloration of facial fur (Cheverud 1996) could potentially affect the markers' detectability, making this species a good candidate for testing the robustness of this methodological approach. At the same time, the complex behavioral and communicative repertoires of cotton‐top tamarins offer the opportunity to evaluate the ability of this technique to capture the variability of facial configurations among a broad range of behaviors.

Our study aimed to understand whether (a) facial gestures could be reliably detected using a deep learning approach, and we predicted that limited effort during the learning phase would allow reliable identification of selected points for quantification of facial expressions; (b) whether gestures associated with vocal signals differed from those shown without phonation, and we predicted that the frames in which the participants were emitting vocalizations would be noticeably different from those in which they were not vocalizing. We predict this based on the assumption that changes in the lip area's configuration and the distances between mouth corners, eyes, and nose influenced the markers' distances. Previous studies on different primate species (Hauser and Ybarra 1994; Ghazanfar 2013; Carugati et al. 2025) already observed that facial expressions are distinctive when associated with the emission of vocalizations. Finally, (c) we also aimed to understand whether facial gestures differed across behavioral contexts, and we predicted that the cotton‐top tamarins' facial gestures varied depending on the social and behavioral context, as observed for chimpanzees (Parr et al. 2005) and black crested macaques (Clark et al. 2020).

## 2 Materials and Methods

### 2.1 Data Collection

We recorded the faces of five individuals of *S. oedipus* hosted at the Zoological and Botanical Park of Mulhouse (Alsace, France) between April 27, 2023, and July 23, 2023. We made all the videos using a 4 K Panasonic HC‐X2000, a professional‐grade camera capable of recording at 60 frames per second (fps), making this tool suitable for capturing the rapid movements of our target subjects. We used an opportunistic approach to conduct the recordings, observing the individuals outside the enclosures from 9:00 a.m. to 5:00 p.m. We filmed the subjects' faces whenever visible and at a suitable distance (2−10 m). We collected 2501 clips with a mean duration of 21.03 ± 13.88 s. We report the distribution of the clips across the individuals in Supporting Information S1: Table S1.

### 2.2 Data Preparation and DLC Model Development

We initially processed the videos with the open‐source software BORIS (Friard and Gamba 2016) to identify and extract high‐quality clips featuring a single, clearly visible face without any interposed objects standing between the animal and the camera. We annotated each clip to indicate whether the subject was emitting a vocalization (*voiced*, “vo”) or not (*unvoiced*, “un”). We classified each clip according to the predominant behavioral context exhibited by the subject, using six macro‐categories—*Feeding* (Fe), *Locomotion* (Lo), *Resting* (Rs), *Scanning* (Sc), *Social Activity* (Sa), *Other Activity* (Oa)—as outlined in the ethogram defined by Edwards et al. (2010). We included Yawning (Yw) as a separate category since its facial configuration shows through a manifest and highly distinct facial display. We also retained the category “vo” as an independent behavioral class to investigate which behaviors might be most easily mistaken for a facial configuration associated with vocalization.

We obtained 3185 clips, with an average duration of 8.68 ± 8.91 s. We reported their distribution among the categories in Supporting Information S1: Table S2. To standardize and reduce the video size, we used the FFMPEG framework (Tomar 2006) to convert the videos to a resolution of 960 × 540 pixels.

Subsequently, we imported all the processed recordings to the open‐source software DeepLabCut (version 2.3.8), hereafter DLC (Mathis et al. 2018; Nath et al. 2019). We used the function extract_frames to sample 10 frames from each clip. Through the DLC graphical interface, we manually labeled the position of a custom set of 13 key points (also referred to as landmarks) designed to mark critical areas for efficiently describing facial movements. We selected the points from the primate_face model from the DeepLabCut Animal Zoo (provided by Claire Witham at the Centre for Macaques, MRC Harwell, UK), which comprises 55 key points delineating the facial features of the rhesus macaque (*Macaca mulatta*). Given the involvement of multiple operators (D.C.G. and F.C.) in the labeling phase, we assessed inter‐rater reliability using the intraclass correlation coefficient (ICC; Shrout and Fleiss 1979) on 200 frames extracted from 20 randomly selected clips, revealing excellent agreement (0.978 < ICC < 0.988) between labelers. We partitioned the coordinates of the labeled key points into training (95%) and test (5%) data sets to develop the DLC model. Among the different algorithms available within DLC, we opted for the ResNet‐50 convolutional neural network (Insafutdinov et al. 2016; He et al. 2016), employing default parameters and progressively increasing the number of iterations in each run (100,000, 200,000, 400,000, 800,000, 1,030,000). We performed two shuffles (Model 1 and Model 2) and applied a 0.6 probability cut‐off. We selected the model with better performance (expressed through a lower root mean square error, hereafter RMSE) for its application in further analysis. We employed the model chosen to analyze all the videos and generate files.csv reporting predicted coordinates of the key points for each frame. Through this process, we also generated the labeled videos, making it possible to visually verify the tracking ability of the model on our video recordings. Examples of labeled videos are available in the Videos S1−S3.

### 2.3 Novel Videos Analysis

To assess the ability of the DLC model to generalize to unseen videos, we randomly selected 20 clips not included in the training/testing sets. Considering the opportunistic approach of the data collection, each clip could consist of a different degree of visual “noise,” expressed as the difference in the position/distance of the subject, in the lighting, or in the recording environment. We used the DLC graphical interface to sample 10 randomly selected frames, and we manually labeled the position of our landmarks. We used the best‐performing DLC model to predict the position of the key points in those frames. Subsequently, we computed the mean euclidean absolute distance (MEAD) between the coordinates of the manually labeled and predicted landmarks only when the estimated points showed a likelihood higher than the *p*‐cutoff (0.6).

### 2.4 Data Normalization and Classification Algorithms

We performed preprocessing steps to the predicted coordinates before the classification analysis. First, we integrated into our pipeline elements derived from the facial alignment process, a technique that allows us to reduce the geometric variation of faces through affine transformations, such as point rotation (Feighelstein et al. 2022; Wei et al. 2020; Morozov et al. 2021). We adapted this approach to our study, computing the angle of rotation between the coordinates of the two inner eye parts (RightEye_Inner‐LeftEye_Inner). Then, we used a custom‐made Python script (version 3.8.3) to rotate all the landmarks' coordinates, ensuring that the line connecting these key points was horizontal (180°).

**Figure 1:**
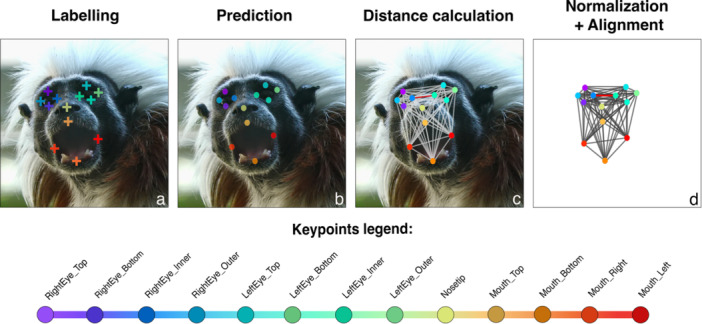
The set of key points used for training the model on DLC plotted on a frame showing a cotton‐top tamarin during the emission of a vocalization (Scream): labeled by the human operator (a), predicted by the DLC model (b), converted in distance matrix (c), aligned and normalized (d). The red line in (c) and (d) indicates the distance selected for normalization.

As a second step, we employed a Python script to transform the aligned coordinates of the predicted key points into distance matrices computed as the Euclidean distance between each pair of points. From a total of 697,723 matrices, we selectively retained only those with all the key points predicted with a likelihood higher than the cut‐off (0.6), resulting in 129,465 fully‐crossed matrices. Before proceeding further, we normalized all distances using the distance between the RightEye_Inner and LeftEye_Inner key points since this measure remains constant regardless of facial movements. This standardization helped to mitigate the variability related to differences according to subjects' distances and positions from the recording camera, as shown in Figure S1. We imported all the normalized matrices into the R software (R Core Team 2021, version 4.1.2). We tabulated to construct a data frame comprising 77 variables corresponding to the number of nonredundant or constant (i.e., 0 and 1) pairs of distances. The main steps of the pipeline are summarized in Figure 1.

Given the potential inherent correlations between the facial distance measurements, we conducted a correlation analysis using the R package “stats” (version 4.1.2). We then ranked variables by their correlation coefficients and excluded those with correlations exceeding 0.75. This process typically resulted in a set of variables in which we retained one representative from each highly correlated group while removing the others. By discarding irrelevant or redundant variables, this feature selection method substantially enhances the performance of neural networks, improving both efficiency and training time in deep learning models (Tirelli and Pessani 2011; Cai et al. 2018). We retained 16 variables from this procedure in the Supporting Information S1: List_S1.

To evaluate the ability to discriminate between voiced and unvoiced facial configuration automatically, we subjected the selected variables to three competitive machine learning techniques: a multi‐layer perceptron (MLP), a support vector machine (SVM), and a random forest classifier (RFC). Given disparities in the number of vocalized and not‐vocalized frames, we subsampled an equal number (*N* = 3000) of instances for each class in every run. Each algorithm ran 100 times. We performed the MLP using the mlp function (package RSNN version 0.4.14), specifying learnFuncParams = 0.1 and maxit = 100 (Bergmeir and Benítez 2012). For the SVM, we utilized the SVM function (package e1071 version 1.7.9), exploring gamma values ranging from 0.005 to 0.050 (0.005, 0.010, 0.015, 0.020, 0.025, 0.030, 0.035, 0.040, 0.045, 0.050), cost values ranging from 10^−8^ to 10°, and coef0 values of 0.1, 1, and 10. We opted for a polynomial kernel with degree = 2, selecting the best gamma, cost, and coef0 parameters based on tuning results (Dimitriadou et al. 2006). We conducted the Random Forest classification in R using the randomForest function (package randomForest), specifying *N* trees = 500 and *N* variables at each split = 3. We trained each algorithm using 70% of each subsample and tested it on the remaining 30%. We extracted the mean and standard deviation correct classification rates (CCR). After assessing distribution using the Shapiro−Wilk test (Shapiro and Wilk 1965), we applied a paired *t*‐test (De Winter 2019) to verify if there were significant differences in the performances of each technique.

We applied the same methodological approach to assess the potential to discriminate among facial gestures occurring during various behavioral contexts automatically. Considering substantial numerical differences among the behavioral classes, we adjusted the re‐sampling size for each run to match the class with the smallest number of cases (the category Yawning), resulting in a subsample size of *N* = 200. Again, all the algorithms ran 100 times.

## 3 Results

**Figure 2:**
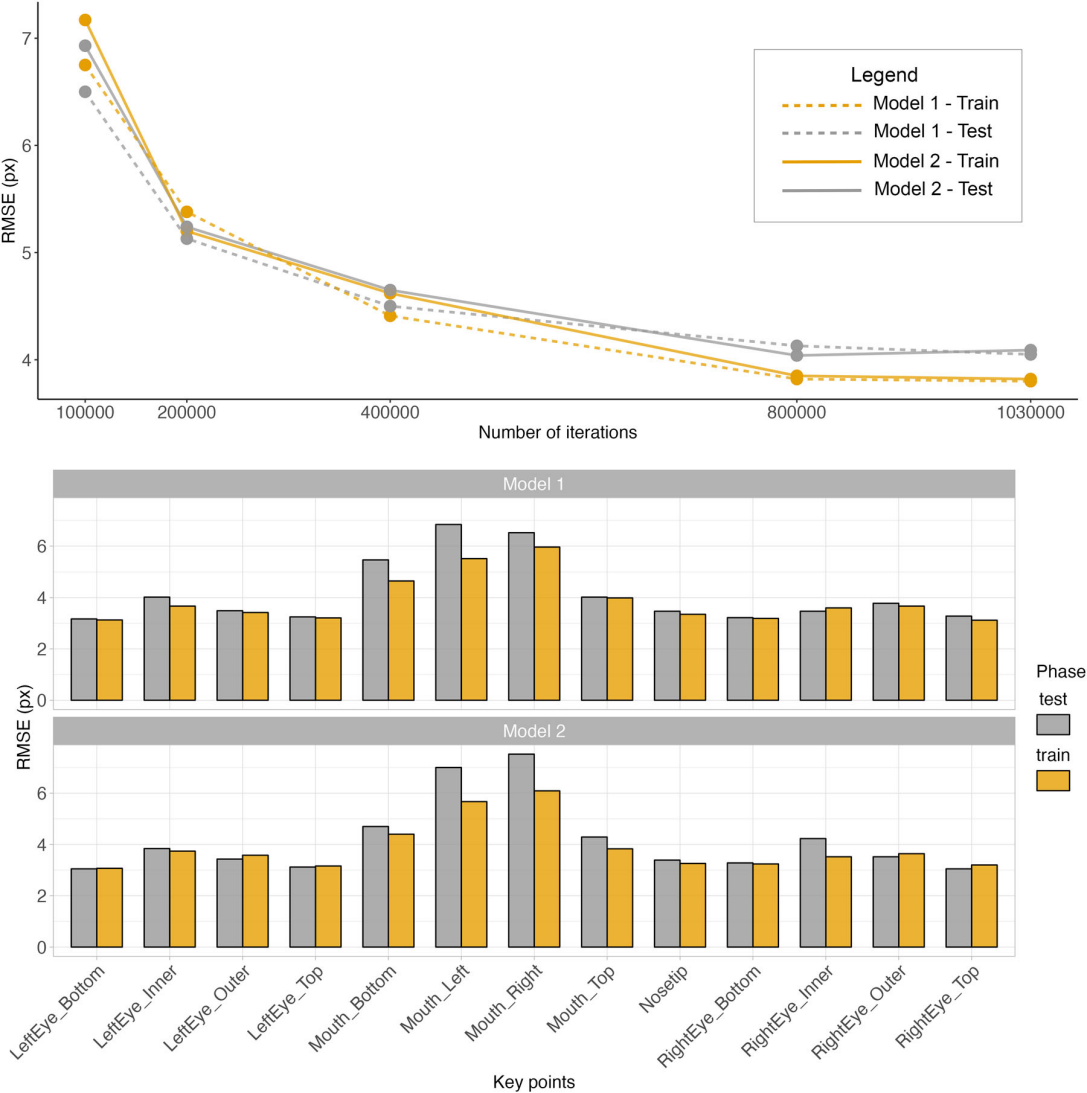
DLC models' RMSE for each trained model (Model 1 and Model 2) at different numbers of iterations and across each landmark.

We developed two robust DLC models that showed progressively lower values of RMSE with an increasing number of iterations, reaching a performance plateau at approximately 800,000 iterations. Model 2 (at 1,030,000 iterations) achieved the lowest RMSE (train: 3.80; test: 4.05) and was selected for further analysis. Notably, we observed remarkable differences in performance across each landmark, with key points positioned around the mouth—particularly at its sides—exhibiting higher errors in both models. Performance metrics for all trained models and each landmark are summarized in Figure 2. We provided readers with examples of labeled videos using the developed models in the Videos S1–S3.

The selected model demonstrated promising results in generalizing the key‐point predictions to unseen images extracted from videos that we did not use to create the train or test sets. The comparison between manually labeled and predicted landmarks revealed low error levels, quantified as MEAD, estimated at 3.93 ± 2.52 pixels. However, as observed for the models' performances, MEAD values varied across key points, with higher values observed for mouth landmarks, as detailed in Table 1. We provided examples of novel videos labeled with the DLC model in the Videos S4−S6.

**Table 1:** Model performance across facial key points within novel videos. The number of detections that exceed the *p*‐cutoff is reported for each landmark.

Key‐points	MEAD ± SD	*N* detections
RightEye_top	3.30 ± 2.21	160
RightEye_Bottom	3.79 ± 2.73	161
RightEye_Inner	3.81 ± 2.29	154
RightEye_Outer	3.68 ± 2.56	159
LeftEye_top	3.56 ± 1.98	132
LeftEye_Bottom	3.46 ± 2.07	135
LeftEye_Inner	3.89 ± 2.46	99
LeftEye_Outer	3.44 ± 1.98	130
Nosetip	3.55 ± 2.01	150
Mouth_Top	4.10 ± 2.54	164
Mouth_Bottom	4.38 ± 2.94	160
Mouth_Right	5.15 ± 3.25	132
Mouth_Left	4.92 ± 3.67	97

Abbreviation: MEAD = mean absolute Euclidean distance.

**Figure 3:**
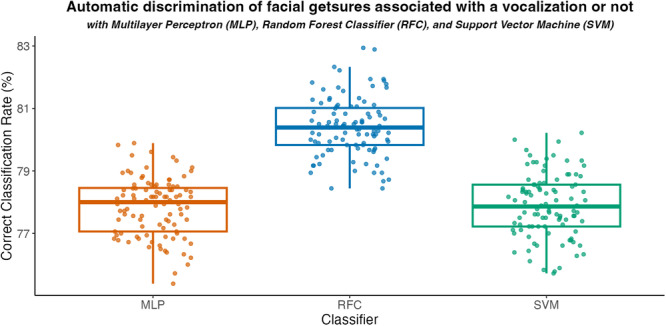
Correct classification rates for the test set of each classifier.

All the machine learning techniques we used to distinguish facial gestures associated or not with the emission of a vocalization performed above the chance threshold (50%). The best‐performing algorithm was RFC, with a correct classification rate of 80.40 ± 0.94%, while MLP and SVM had similar results at 77.85 ± 0.93% and 77.89 ± 1.01%, respectively, as shown in Figure 3. Paired *t*‐tests displayed significant differences between RFC and the other algorithms (MLP‐RFC: *t* = −25.54, df = 99, *p *< 0.01; RFC‐SVM: *t* = −37.44, df = 99, *p *< 0.01), while no significant difference emerged between MLP and SVM (MLP‐SVM: *t* = −0.33 df = 99, *p *= 0.74). The mean and standard deviation of the machine learning metrics (accuracy, precision, recall, and F1 score) are reported in Table 2.

**Table 2:** Mean and standard deviation of accuracy, precision, recall, and F1 score of each ML algorithm (train and test) for discriminating facial gestures related to vocalization emission.

Metric (mean ± SD)	MLP		SVM		RFC	
	Train	Test	Train	Test	Train	Test
Accuracy	0.80 ± 0.01	0.78 ± 0.01	0.79 ± 0.01	0.78 ± 0.01	0.80 ± 0.01	0.80 ± 0.01
Precision	0.79 ± 0.02	0.77 ± 0.02	0.86 ± 0.01	0.84 ± 0.02	0.78 ± 0.01	0.84 ± 0.02
Recall	0.82 ± 0.03	0.80 ± 0.04	0.76 ± 0.01	0.75 ± 0.02	0.84 ± 0.01	0.78 ± 0.02
F1 score	0.80 ± 0.01	0.78 ± 0.01	0.81 ± 0.01	0.79 ± 0.01	0.81 ± 0.01	0.81 ± 0.01

Regarding the ability to automatically discriminate among facial gestures associated with different behavioral contexts, we observed higher‐than‐chance results for all the applied classifiers that showed CCR that overcame 12.5% (corresponding to the chance threshold of an 8‐level classification). The technique that showed higher performances was again RFC, with a correct classification rate of 53.84 ± 2.27%, followed by SVM (35.57 ± 2.35%) and MLP (33.59 ± 2.03%). Table 3 details the metrics relative to each algorithm.

**Table 3:** Mean and standard deviation of accuracy, precision, recall, and F1 score of each ML algorithm (train and test) for discriminating facial gestures related to different behavioral contexts.

Metric (mean ± SD)	MLP		SVM		RFC	
	Train	Test	Train	Test	Train	Test
Accuracy	0.37 ± 0.01	0.34 ± 0.02	0.39 ± 0.02	0.36 ± 0.02	0.54 ± 0.01	0.54 ± 0.04
Precision	0.38 ± 0.02	0.34 ± 0.02	0.44 ± 0.02	0.41 ± 0.03	0.54 ± 0.01	0.54 ± 0.02
Recall	0.37 ± 0.01	0.35 ± 0.03	0.39 ± 0.02	0.36 ± 0.02	0.54 ± 0.01	0.54 ± 0.02
F1 score	0.36 ± 0.02	0.32 ± 0.02	0.39 ± 0.02	0.36 ± 0.03	0.53 ± 0.01	0.53 ± 0.02

All paired *t*‐tests showed significant differences among the performances of each algorithm (RFC‐MLP: *t* = −72.811, df = 99, *p* < 0.01; MLP‐SVM: *t* = −7.1146, df = 99, *p* < 0.01; SVM‐RFC: *t* = −73.945, df = 99, *p* < 0.01).

**Figure 4:**
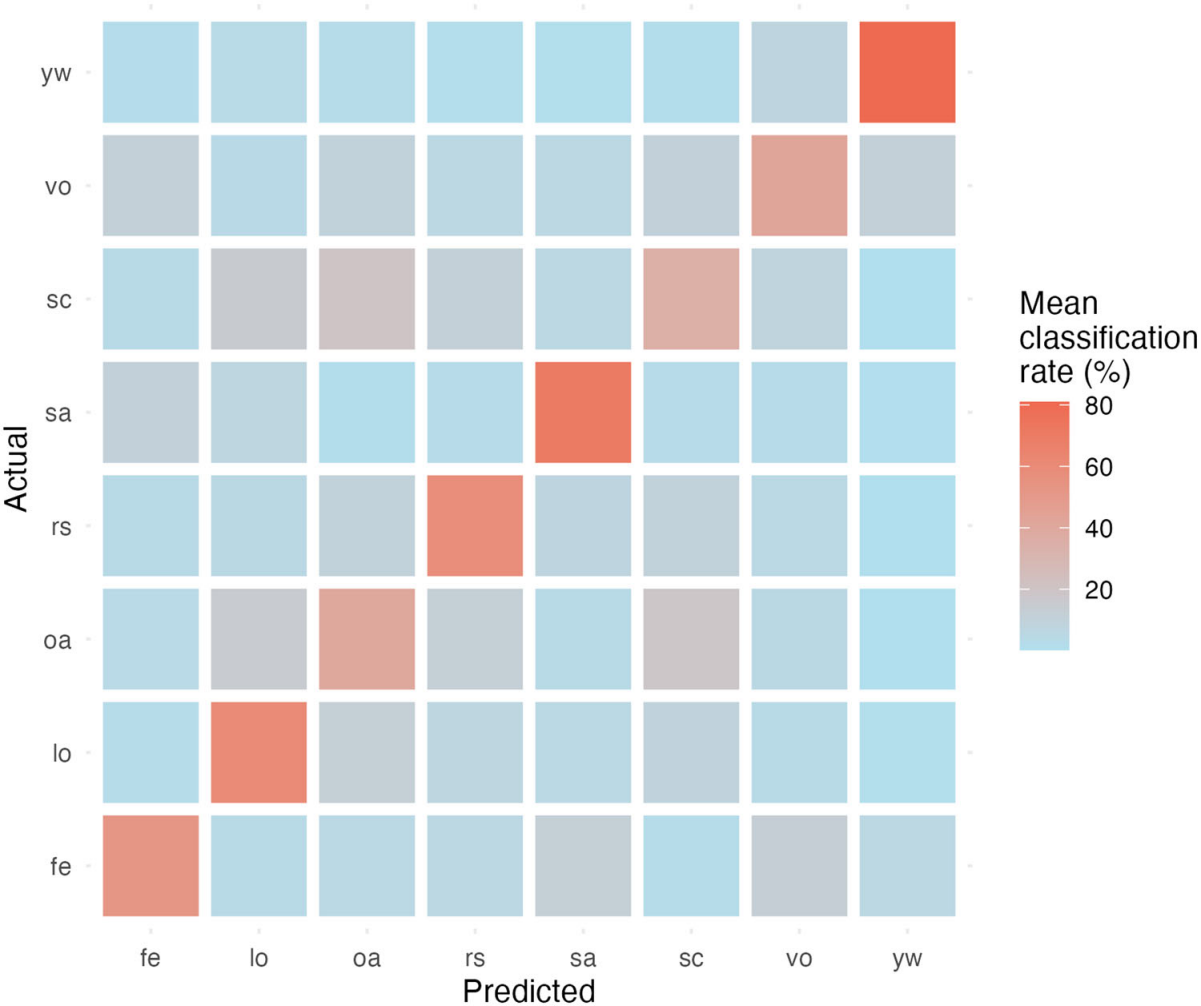
Heatmap of the confusion matrix that explains the classification percentages for each behavioral category in the RFC test. The behavioral categories are *Vocalization* (vo), *Feeding* (fe), *Locomotion* (lo), *Resting* (rs), *Scanning* (sc), *Social Activity* (sa), *Other Activity* (oa), and *Yawning* (yw).

Focusing on the best classification technique (RFC) results, as previously mentioned, we observed marked differences across the different behavioral contexts despite all the CCRs overcoming the chance threshold in all the levels. Facial gestures associated with a Yawning (yw) were more often correctly classified (81.09 ± 4.58%), followed by *Social activity* (sa: 74.72 ± 6.24%), resting (rs: 59.76 ± 6.22%), and locomotion (lo: 55.43 ± 6.50%). We visualized the confusion matrix resulting from 100 runs of RFC in Figure 4. We reported the mean and standard deviation of the classification rates for each behavioral category in Supporting Information S1: Table S3.

## 4 Discussion

How viable is machine learning for reliable decoding of primate facial gestures? Our study demonstrated the potential of employing deep learning techniques to quantify the facial gestures of small and fast‐moving primate species. We used a markerless pose estimation system to develop a robust model that efficiently predicts the position of a custom set of key points on cotton‐top tamarin faces. From the predicted coordinates, we could automatically discriminate the facial configuration that cooccurs with the emission of a vocalization, overcoming 80% of correct classification. Furthermore, we applied the same methodological approach to classifying the facial configuration associated with different behavioral contexts, achieving higher‐than‐chance classification rates for all the categories.

### 4.1 Detection of Facial Gestures Using a Deep Learning Approach

This work highlights the promising role of deep learning algorithms, particularly DeepLabCut software, in capturing primate facial configuration, also when applied to detect landmarks distributed on the face of a small and fast species. Our model showed RMSE values—the performance metric more widely used to evaluate the reliability in detection—that are consistent with those of DLC models previously developed for primates' markerless pose estimation (Wiltshire et al. 2023; Labuguen et al. 2019, 2021; Lauer et al. 2022; Fuchs et al. 2024; Carugati et al. 2025), supporting the robustness of our model. Moreover, the RMSE related to each key point indicated that points around the mouth, particularly those positioned on its sides, were predicted with less precision than key points marking the eyes and nose. Given that cotton‐top tamarins have dark, fur‐covered faces, we hypothesize that the detected differences can be imputable to the higher uniformity of the area surrounding the lips, especially when the jaws are closed, resulting in a continuous blackish pattern. Conversely, key points distributed around the eyes were predicted more accurately, likely due to the highly distinctive features of these points. These considerations apply not only to the model predictions but also to the labeling phase, where the human labeler's precision can vary based on its ability to distinguish the position of the point in randomly extracted frames. Remarkable differences among key‐points prediction accuracy have also emerged in other DLC models tracking primates (Labuguen et al. 2021; Wiltshire et al. 2023). Notably, the few key points on the face of the target species were the ones with better performances than the other body parts, especially the points positioned in distal areas, such as hips and ankles. Our results strengthen the idea that primate faces represent an optimal target from markerless pose estimation using deep neural networks.

Furthermore, the novel video analysis demonstrated that the model exhibited exceptional generalization in landmark prediction across unseen images, which we did not include in the model development phase. Specifically, our findings revealed significantly lower MEAD values than other DLC models designed for primates (Wiltshire et al. 2023; Carugati et al. 2025). This finding is particularly promising given the challenging characteristics of the target species, such as small size, rapid movements (which increase the likelihood of blurred frames), and a face covered with dark fur. However, we observed variations in MEAD among different key points, particularly with mouth‐side landmarks exhibiting a higher error rate and fewer detections. These disparities were also noted in the study by Carugati et al. (2025), underscoring how landmark positioning and intrinsic features, such as contrast levels around the landmark, contribute to significant fluctuations in prediction accuracy.

These considerations inevitably affect the number of key points selected for model development, representing a potential limitation of this methodological approach. Undeniably, more landmarks can provide a more detailed capture of facial configuration variability, including subtle movements of cheeks, eyebrows, and ears—features critical to facial expressions in many primate species (Waller et al. 2020). However, increasing the number of landmarks requires careful balancing of several factors, particularly considering the intrinsic characteristics of each key point, such as its detectability and position. Priority should be given to landmarks that human operators easily identify during labeling. Additionally, since the workflow presented in this paper relies on a fully crossed set of distances, incorporating more landmarks may reduce the number of frames where all key points meet the cut‐off threshold, limiting the number of usable images. While further future investigation may apply more complex models, this study demonstrates the feasibility of quantifying facial configurations that appeared differentially between behavioral contexts using a simple model based on 13 key points.

### 4.2 Facial Gestures Associated With Vocalizations Differed From Those Unvoiced

The fact that primates articulate their faces in a particular way when vocalizing has been reported in several studies, which included humans (Lyons et al. 1998; Hontanilla and Aubá 2008; Dagnes et al. 2019; Yehia et al. 1998; Yehia et al. 2002), macaques (Hauser et al. 1993; Hauser and Ybarra 1994; Ghazanfar 2013), and lemurs (Favaro et al. 2008; Gamba et al. 2011). In line with the study by Carugati et al. (2025), we wanted to investigate whether we could also detect distinctive gestures for the emission of vocalizations in cotton‐top tamarins. This analysis does not represent an exercise in style but opens the door for applications that address multimodal communication in primates in a modern way. Machine learning techniques enabled the discrimination of facial gestures occurring during a vocalization from those not associated with a vocal emission. We could directly compare our findings with the study on indris (*Indri indri*), diademed sifaka (*Propithecus diadema*), and yellow‐cheeked crested gibbon (*Nomascus gabriellae*). Carugati et al. (2025) found that those species' CCR exceeded 90%. We hypothesized that the differences in performance between *S. oedipus* and the other species investigated so far could be related to different factors. The first concerns the vocal repertoire of cotton‐top tamarins: they produce vocalizations, such as Twitters and Trills, which are emitted with closed or semi‐open mouth (Cleveland and Snowdon 1982), which may not be sufficiently differentiated in the facial configuration to be accurately classified by machine learning algorithms, impacting the overall performance.

Considering that all previously examined species include vocalizations in their repertoire that do not determine a noticeable change in facial configuration (e.g., *I. indri*, Maretti et al. 2010; *Propithecus* spp., Patel and Owren 2012), we can hypothesize that our results align with those of Carugati and colleagues (2025). Specifically, the presence of calls produced without visible facial movements (e.g., nasal calls) may account for the misclassified cases based on the frequency of their occurrence in the recordings containing vocalizations.

On the other hand, we need to consider that indris and gibbons perform a highly distinctive vocal behavior that determines a remarkable modification of the facial configuration (Favaro et al. 2008; Gamba et al. 2011; Koda et al. 2012), namely, song production. Songs are known for lasting way longer than calls (De Gregorio et al. 2022), and considering the extended duration of song emissions and the relative ease of capturing these events during opportunistic data collection, it is likely that the frames extracted from these sequences constitute an important component of the sampled vocal emissions. On the other hand, short calls are more challenging to capture, resulting in a lower representation. We can, therefore, hypothesize that both the composition of the specific vocal repertoire and the proportion of each vocal type included in our training and test data set may influence the performance of the automatic classification, achieving higher results when vocal utterances that determine remarkable changes in orofacial configuration are more often sampled.

An additional factor to interpret our result is related to a type of vocal utterances of the cotton‐top tamarins, the food‐associated calls: in fact, the vocal repertoire of these primates includes two calls (C and D chirps) that are strictly associated with foraging and feeding (Cleveland and Snowdon 1982; Elowson et al. 1991; Roush and Snowdon 1999; Roush 1996). Considering that these vocal emissions have a brief duration and could be integrated with the movement of the jaws during chewing, it is possible that, in some cases, there was a substantial similarity in face configuration. In support of this hypothesis, the confusion matrix from the subsequent analysis reveals that misclassified facial configurations associated with vocalization were most frequently labeled as associated with the category “Fe,” which pertains to feeding behaviors.

### 4.3 Facial Gestures Differed Across Behavioral Contexts

The automatic classification of facial gestures associated with different behavioral contexts showed higher‐than‐chance CCR, indicating a substantial variation of the facial features across behavioral contexts. However, the CCR considerably varied across categories, suggesting that facial configurations have a different degree of context‐specificity. The best predicted facial gestures were the ones associated with a Yawn (CCR = 81.09 ± 4.58%). This result is in agreement with the fact that yawns determine a manifest and unique morphological change in facial appearance, involving a wide mouth opening with jaw‐dropping, often associated with closed eyes and head reclination, as described for different primate species, including geladas (Palagi et al. 2009; Leone et al. 2014), drills (Galotti et al. 2024), and humans (Baenninger 1997). However, it is interesting to notice that the RFC algorithm associated most of the misclassified cases of this class within the category “Vocalization,” confirming the strong relationship between mouth opening and vocal emission: in fact, cotton‐top tamarins can elicit some vocal emissions described with a simultaneous wide opening of the jaws, such as the Normal Long Calls, the Type A Chirps, and the Slicing Screams (Cleveland and Snowdon 1982). We obtained a high value of correct classification (CCR = 74.72 ± 6.24%) also for the facial gestures associated with the context “Social activity,” a macro‐category that includes allogrooming, play, affiliative and sexual behaviors (Edwards et al. 2010). Despite the heterogeneous composition of this category, the analysis showed a high degree of context‐specificity. This result is in agreement with several studies that show how most of the facial gestures were produced preferentially in specific social interactions, highlighting the communicative function of these displays, as shown in red‐capped mangabeys (Aychet et al. 2021), white‐faced capuchins (De Marco et al. 2008), and chimpanzees (Parr et al. 2005).

Another behavioral context whose facial gestures showed a remarkable degree of context specificity was “Resting,” which showed a correct classification rate equal to 59.76 + 6.22%. In our work, this category comprised both resting and sleeping. During these activities, animals can have their eyes open or closed (in resting) or closed for an extended time (sleeping; Allison and Cicchetti 1976). In captive cotton‐top tamarins, resting is the behavior with the highest frequency of occurrence, taking up 32.4% of the total time budget (Edwards et al. 2010). Therefore, closed eyes might have played a role in classifying this facial configuration. Previous work showed how chimpanzees understand the role that eyes (being open or closed) play in attention (Hostetter et al. 2007). Our results align with the idea that faces typically considered “neutral,” such as a resting or sleeping face, may still serve a communicative function, as a “neutral face is conspicuous precisely because of the absence of movement” (Waller et al. 2022).

From a broader—and more etho‐ecological—perspective, this work highlighted how cotton top tamarins modify their facial configurations across different behaviors, showing a remarkable context‐specificity. Similar findings have been documented for chimpanzees (Parr et al. 2005) and black‐crested macaques (Clark et al. 2020), showing how primates can use different facial signals to mediate behavioral interactions. Although the literature on tamarin facial behavior remains limited, this study contributes to understanding the complexity of cotton‐top tamarin facial configurations. Our findings suggest that this species possesses a rich facial variability, with clear distinctions across different behavioral contexts. This highlights the communicative potential of tamarin facial signals and the broader ecological and social significance of such expressions in their natural interactions.

In conclusion, this work showed the potential of using markerless pose estimation algorithms to quantitatively describe primate facial gestures, even in challenging species such as cotton‐top tamarins. Automatic methods dramatically reduce the time required for extracting information from video recordings, and they can represent a revolutionary tool for animal behavior studies. Although the complete automation of extracting and classifying behavioral information remains a distant goal, this study represents a significant initial step in that direction. It offers a novel methodological approach and delivers promising results regarding its potential application, paving the way for multiple scenarios in animal communication and behavior studies.

## Ethics Statement

The noninvasive methods used for this study's data collection adhere to the American Society of Primatologists (ASP) “Principles for the Ethical Treatment of Non‐Human Primates.” The Mulhouse Zoo reviewed and approved the data collection protocol.

## Conflicts of Interest

The authors declare no conflicts of interest.

## Supporting information

Supporting information.

Supporting information.

Supporting information.

Supporting information.

Supporting information.

Supporting information.

Supporting information.

Supporting information.

## Data Availability

The data supporting this study's findings are available from the corresponding author upon reasonable request.
