# Supplementary material for: Quantifying Facial Gestures Using Deep Learning in a New World Monkey
Source: Am J Primatol. 2025 Feb 28;87(3):e70013. doi: 10.1002/ajp.70013 (PMC11869534; doi:10.1002/ajp.70013)
Supplement: Supplementary file 1 — Supporting information. [file AJP-87-e70013-s003.docx]

**SUPPLEMENTARY MATERIAL**

**TABLE S1**

| **Presence of vocal emission** | **Behavioral context** | **Individuals** | | | | | **Total** |
| --- | --- | --- | --- | --- | --- | --- | --- |
|  |  | **Alita** | **Limon** | **Maeva** | **Wloc** | **Woop** |  |
| **vo** | **vo** | 228 | 80 | 50 | 192 | 77 | 627 |
|  | **fe** | 101 | 54 | 107 | 100 | 122 | 484 |
|  | **lo** | 6 | 9 | 0 | 7 | 6 | 28 |
|  | **oa** | 32 | 65 | 52 | 37 | 124 | 310 |
| **un** | **rs** | 16 | 10 | 13 | 18 | 15 | 72 |
|  | **sa** | 6 | 17 | 0 | 8 | 9 | 40 |
|  | **sc** | 163 | 205 | 160 | 203 | 198 | 929 |
|  | **yw** | 2 | 2 | 1 | 2 | 4 | 11 |
| **Total** | | **554** | **442** | **383** | **567** | **555** | **2501** |

**Table S1**. Distribution of the original videos recorded across the behavioral contexts and the individuals.

**TABLE S2**

| **Presence of vocal emission** | **Behavioral context** | **Individuals** | | | | | **Total** |
| --- | --- | --- | --- | --- | --- | --- | --- |
|  |  | **Alita** | **Limon** | **Maeva** | **Wloc** | **Woop** |  |
| **vo** | **vo** | 228 | 96 | 67 | 248 | 95 | 786 |
|  | **fe** | 110 | 77 | 138 | 114 | 132 | 571 |
|  | **lo** | 8 | 18 | 1 | 13 | 20 | 60 |
|  | **oa** | 67 | 102 | 77 | 57 | 155 | 458 |
| **un** | **rs** | 17 | 28 | 10 | 19 | 11 | 85 |
|  | **sa** | 2 | 13 | 0 | 9 | 4 | 28 |
|  | **sc** | 214 | 257 | 179 | 287 | 247 | 1184 |
|  | **yw** | 2 | 2 | 3 | 2 | 4 | 13 |
| **Total** | | **700** | **593** | **475** | **749** | **668** | **3185** |

**Table S2.** Distribution of the clips across the behavioral contexts and the individuals.

**List_S1**

**List variables submitted to ML algorithms**

- RightEye_Outer-RightEye_Top ("X4.1")
- LeftEye_Top-RightEye_Top ("X5.1")
- LeftEye_Bottom-RightEye_Top ("X6.1")
- RightEye_Outer-RightEye_Bottom ("X4.2")
- LeftEye_Top-RightEye_Inner ("X5.3")
- Nosetip-RightEye_Inner ("X9.3")
- Mouth_Top-RightEye_Inner ("X10.3")
- Mouth_Bottom-RightEye_Inner  ("X11.3")
- Mouth_Left-RightEye_Inner ("X13.3")
- LeftEye_Outer-LeftEye_Top ("X8.5")
- LeftEye_Inner-LeftEye_Bottom ("X7.6")
- Nosetip-LeftEye_Inner ("X9.7")
- Mouth_Top-Nosetip ("X10.9")
- Mouth_Right-Nosetip ("X12.9")
- Mouth_Left-Mouth_Top ("X13.10")
- Mouth_Left-Mouth_Right ("X13.12")

**TABLE S3**

|  | **co** | **fe** | **lo** | **oa** | **rs** | **sa** | **sc** | **yw** |
| --- | --- | --- | --- | --- | --- | --- | --- | --- |
| **co** | 44.09 + 6.11 | 16.74 + 5.26 | 2.43 + 1.99 | 4.45 + 2.51 | 3.65 + 2.74 | 2.71 + 2.24 | 8.22 + 3.91 | 5.03 + 3.42 |
| **fe** | 16.83 + 5.56 | 43.29 + 5.89 | 2.31 + 2.22 | 4.03 + 2.49 | 1.54 + 1.55 | 8.60 + 4.08 | 4.76 + 2.91 | 2.99 + 2.22 |
| **lo** | 3.3 + 2.3 | 4.00 + 2.81 | 55.43 + 6.5 | 11.49 + 4.46 | 6.78 + 3.35 | 6.28 + 3.01 | 10.94 + 3.74 | 3.11 + 2.38 |
| **oa** | 7.87 + 3.53 | 7.54 + 3.02 | 11.34 + 4.69 | 41.24 + 6.70 | 9.33 + 4.65 | 1.76 + 2.01 | 20.21 + 5.04 | 3.26 + 2.11 |
| **rs** | 4.26 + 3.07 | 5.48 + 3.35 | 7.14 + 3.40 | 11.75 + 4.12 | 59.76 + 6.22 | 1.51 + 1.56 | 13.49 + 4.97 | 1.48 + 1.56 |
| **sa** | 4.41 + 2.56 | 10.08 + 3.76 | 9.23 + 3.85 | 7.48 + 3.57 | 8.54 + 3.49 | 74.72 + 6.24 | 7.82 + 3.63 | 1.15 + 1.27 |
| **sc** | 11.8 + 4.63 | 8.24 + 3.76 | 10.65 + 4.37 | 18.54 + 5.15 | 9.87 + 4.38 | 3.46 + 2.87 | 33.03 + 6.67 | 1.89 + 1.71 |
| **yw** | 7.44 + 3.31 | 4.63 + 2.60 | 1.47 + 1.48 | 1.02 + 1.27 | 0.52 + 0.88 | 0.98 + 1.58 | 1.53 + 1.65 | 81.09 + 4.58 |

**Table S3.** Confusion matrix reporting mean and standard deviation of classification rates (%) for each behavioral category obtained with the RFC algorithm.
