# Supplementary figures and images for: Quantifying Facial Gestures Using Deep Learning in a New World Monkey
Source: Am J Primatol. 2025 Feb 28;87(3):e70013. doi: 10.1002/ajp.70013 (PMC11869534; doi:10.1002/ajp.70013)

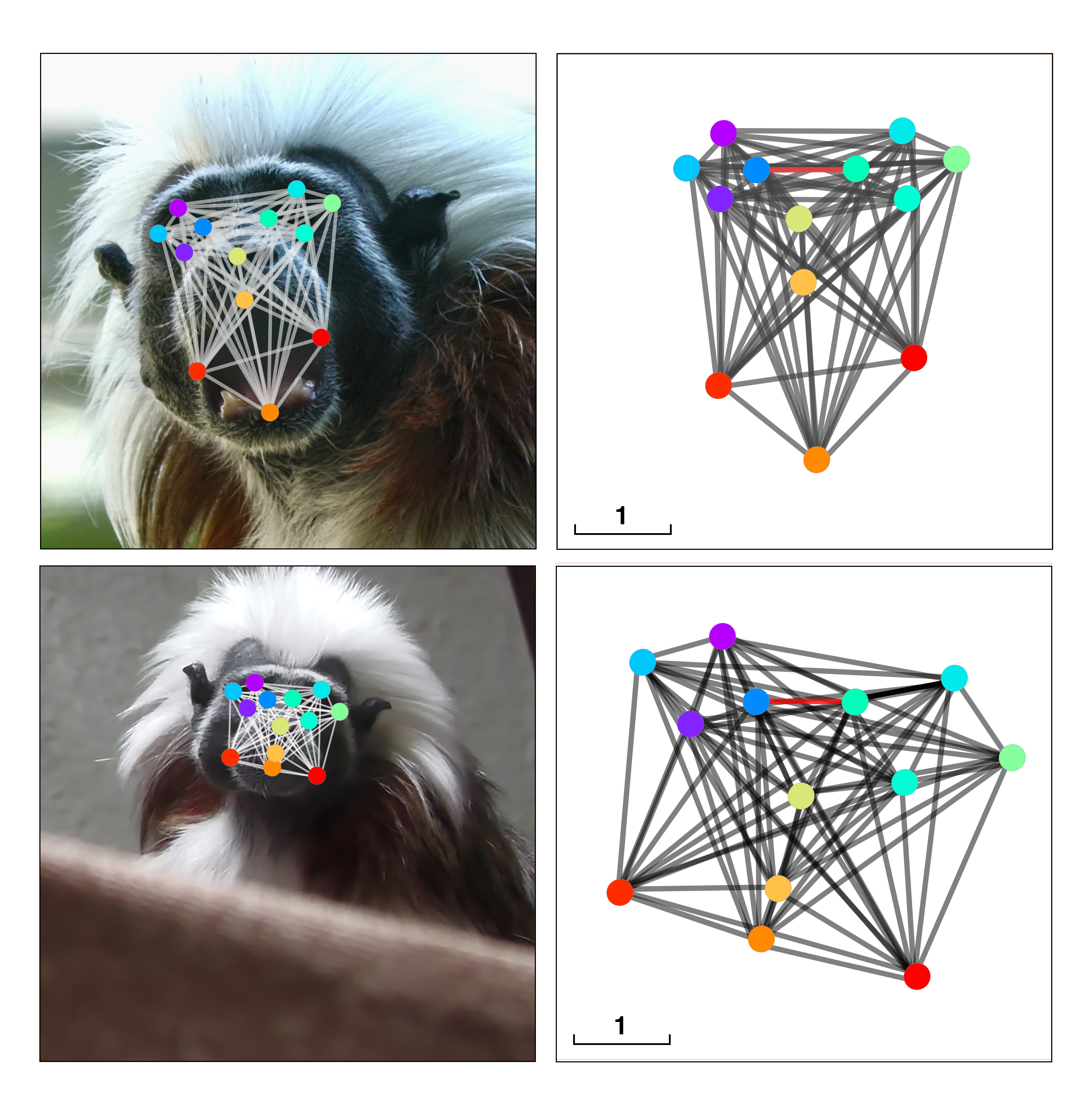

Supplement: Supplementary file 8 — Supporting information. [file AJP-87-e70013-s002.png]
